# Coupling Immunoprecipitation with Multiplexed Digital PCR for Cell-Free DNA Methylation Detection in Small Plasma Volumes of Early-Onset Colorectal Cancer

**DOI:** 10.1021/acs.analchem.5c01361

**Published:** 2025-05-17

**Authors:** Truong T. Truong, Klara Mikloska, Judith Sum, Martina Oberländer, Nikolas von Bubnoff, Lea Christiansen, Sebastian Tornow, Stefanie Derer, Florian Janke, Holger Sültmann, Sebastian Zeissig, Michael Linnebacher, Clemens Schafmayer, Michael Lehnert, Tobias Hutzenlaub, Nils Paust, Peter Juelg

**Affiliations:** † 199773 Hahn-Schickard, 79110 Freiburg, Germany; ‡ Laboratory for MEMS Applications, IMTEK—Department of Microsystems Engineering, 9174 University of Freiburg, 79110 Freiburg, Germany; § Interdisciplinary Center for Biobanking-Lübeck (ICB-L), University Medical Center Schleswig-Holstein, 23562 Lübeck, Germany; ∥ Department of Hematology and Oncology, University Medical Center Schleswig-Holstein, 23538 Lübeck, Germany; ⊥ European Liquid Biopsy Society (ELBS), 20246 Hamburg, Germany; # Institute of Nutritional Medicine, University Medical Center Schleswig-Holstein, 23562 Lübeck, Germany; ¶ Division of Cancer Genome Research, German Cancer Research Center (DKFZ), 69120 Heidelberg, Germany; ∇ Translational Lung Research Center (TLRC), Member of the German Center for Lung Research (DZL), 69120 Heidelberg, Germany; ○ National Center for Tumor Diseases (NCT), 69120 Heidelberg, Germany; ⧫ Department of Internal Medicine A, University Medical Center Greifswald, 17475 Greifswald, Germany; †† Biobank Rostock, Clinic of Surgery, Rostock University Medical Center, 18057 Rostock, Germany; ‡‡ Clinic of Surgery, Rostock University Medical Center, 18057 Rostock, Germany

## Abstract

Colorectal cancer (CRC) remains a major global health challenge, with an increasing incidence of early-onset cases among young adults. Targeted analysis of cell-free DNA (cfDNA) methylation in blood has emerged as a promising minimally invasive diagnostic approach. While digital PCR (dPCR) offers high sensitivity and low turnaround times, conventional bisulfite-based dPCR assays require large plasma volumes due to cfDNA degradation, limiting clinical feasibility. To overcome this limitation, we developed a bisulfite-free, low-plasma-volume assay by coupling cell-free methylated DNA immunoprecipitation (cfMeDIP) with multiplexed dPCR for methylation detection. Assays were designed for CRC targets based on publicly available bisulfite-based plasma data and optimized for native, bisulfite-untreated cfDNA. The cfMeDIP-dPCR assays were first developed and optimized on circulating tumor DNA surrogates derived from HCT116 cells and subsequently validated in a pilot study, including 32 early-onset CRC (EO-CRC) patients and 29 non-CRC individuals. Methylation ratios, defined as the proportion of methylated to total cfDNA copies per marker, served as a diagnostic indicator. Three out of four selected markers (*SEPT9*, *KCNQ5*, and *C9orf50*) were successfully adapted, with significantly higher methylation ratios (*p* ≤ 0.001) in the EO-CRC cohort. *KCNQ5* demonstrated the highest diagnostic performance, achieving an 85% sensitivity at a 90% specificity, with methylation ratios correlating with the tumor stage. This study presents the first cfMeDIP-dPCR approach, demonstrating its potential as a sensitive liquid biopsy assay. Requiring only 0.5 mL of plasma, i.e., more than 20 times less than a sensitivity-matched bisulfite-based assay, cfMeDIP-dPCR facilitates clinical implementation for CRC and other diseases with epigenetic signatures.

## Graphical abstract

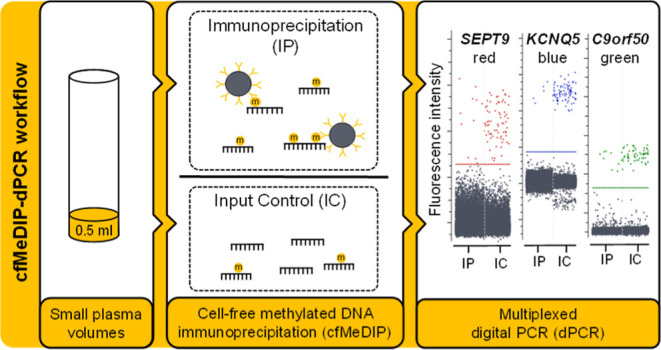

## Introduction

In recent years, liquid biopsies have gained significant traction as minimally invasive methods for the detection of diseases. Particularly in cancer diagnostics, the testing of cancer-specific genetic or epigenetic alterations in cell-free DNA (cfDNA) is increasingly implemented in clinical practice.^1,2^ The cfDNA originating from tumors, known as circulating tumor DNA (ctDNA), is released into the bloodstream or other body fluids, such as urine, saliva, stool, or cerebrospinal fluid.^3–5^ Hence, liquid biopsies offer several advantages over traditional tissue biopsies: They enable a more comprehensive profile of the cancer by capturing its intratumoral heterogeneity, allow repeatable sample collection for longitudinal disease monitoring, reduce patient risk for vulnerable individuals,^6^ and reach higher patient compliance, with blood being the preferred sample type.^7^

Despite these promising benefits, liquid biopsy faces challenges that limit its widespread clinical application. In particular, the sensitivities of such tests are ultimately constrained by the physical prevalence of the target analyte in blood circulation. While increasing plasma volumes could improve test sensitivity, this approach conflicts with the goal of maintaining patient compliance. In this context, cfDNA methylation detection offers unique advantages over cfDNA mutation detection. Compelling evidence suggests that the GC-rich^8^ and hypermethylated^9^ fraction of cfDNA tends to persist longer in circulation compared to their AT-rich and unmethylated counterparts. Additionally, methylation changes occur early in tumorigenesis, making them ideal for early detection.^10^

Technologies widely used for DNA methylation analysis in liquid biopsy diagnostics primarily rely on two detection methods: Next-generation sequencing (NGS) and PCR techniques. Workflows in combination with NGS enable four distinct principles for methylation detection: (i) restriction enzyme-based methods were historically the first approaches for methylation detection^11^ and were shown with cfDNA to accurately diagnose colorectal and lung cancer.^12^ (ii) Conversion-based methods provide base-pair resolution, with chemical bisulfite conversion still regarded as the gold standard for methylation detection.^13^ Bisulfite sequencing (bisulfite-seq) has been widely used with cfDNA for multicancer detection and tissue-of-origin localization.^14–16^ More recently, enzymatic methyl-sequencing (EM-seq), which relies on enzyme-based conversion, has demonstrated potential in distinguishing hepatocellular carcinoma (HCC) and non-HCC individuals.^17^ (iii) Enrichment-based methods rely on the capturing of methylated DNA using either antibodies with high affinity to methylated 5′-carbon of cytosine (methylated DNA immunoprecipitation or short MeDIP) or methyl-CpG-binding domain (MBD).^18,19^ Both MeDIP and MBD have been optimized for cfDNA inputs of 10 ng or lower, amounts typically found in 1 mL of plasma.^20^ These protocols, termed cfMBD-seq and cfMeDIP-seq, were employed to identify patterns of differentially methylated regions (DMRs) across several cancers.^21,22^ And last, (iv) direct methylation calling using nanopore sequencing has been demonstrated as an alternative without requiring extensive pretreatment for the analysis of cfDNA methylomes in cancer patients.^23,24^

While sequencing remains critically important for generating reference methylomes and advancing our understanding of biology and disease, its high costs and lengthy turnaround times pose considerable challenges for routine clinical application. In contrast, digital PCR (dPCR), particularly when combined with bisulfite conversion or restriction enzyme-based approaches, has shown great promise for clinical implementation.^25–27^ However, both NGS- and PCR-based methylation detection methods face limitations associated with bisulfite conversion and restriction enzyme-based approaches, which are exacerbated when applied to cfDNA in a clinical setting. Bisulfite conversion, which most commercial methylation detection assays rely on, leads to significant degradation of the already scarce cfDNA during bisulfite treatment.^28^ Consequently, it requires high plasma volumes of up to 16 mL to ensure sufficient input for analysis.^26,29,30^ In addition, the severely fragmented nature of cfDNA drastically reduces the number of available recognition sites for restriction enzyme-based methods.^31^ On the other hand, enrichment-based methods, such as the cfMeDIP protocol, benefit from fragmentation for efficient pull-down.^32^ Since its development, cfMeDIP-seq has shown promising results across various cancer types and applications including tumor marker identification, healthy population screening, tumor classification, as well as longitudinal tumor monitoring.^33–39^ However, for widespread clinical adoption, it is crucial to consider analysis methods following cfMeDIP sample preparation that are less costly and time-intensive than NGS. Intriguingly, while conventional MeDIP combined with quantitative PCR (qPCR) has been explored in prenatal diagnostics,^40,41^ no studies to date have coupled the liquid biopsy-adapted cfMeDIP sample preparation with sensitive multiplexed dPCR analysis.

In this study, we aim to fill this gap by developing and demonstrating a cfMeDIP-dPCR assay for the detection of methylated cfDNA markers in small plasma volumes, which takes advantage of (i) conversion- and restriction enzyme-free cfMeDIP sample preparation and (ii) cost- and time-efficient dPCR analysis. Rather than identifying new methylation markers, we focused on leveraging promising colorectal cancer (CRC)-specific markers from existing bisulfite-based studies and adapting them for use in a dPCR-panel compatible with the cfMeDIP protocol.

We opted for CRC as the model for this study, given its well-characterized methylation markers and significant global impact. CRC is the third most frequently diagnosed type of cancer and the second leading cause of cancer-related deaths worldwide.^42^ While the incidence of CRC has stabilized in high-income countries for individuals 50 years and older, there has been a concerning rise in early-onset CRC (EO-CRC), defined as CRC occurring in individuals under 50 years of age.^43,44^ Early detection is crucial for improving patient outcomes at all ages, and methylated cfDNA markers have shown great potential for minimally invasive CRC screening.^30^

## Experimental Section

### Study Design

This study pursued two primary objectives: (i) technically, to select and transfer CRC-related methylation markers, previously validated using bisulfite-based methods, to a bisulfite-free, multiplexed cfMeDIP-dPCR assay. This approach aimed to leverage the advantages of the cfMeDIP-dPCR assay while addressing the challenges inherent in transitioning from PCR systems designed for bisulfite-converted targets to systems that target the native sequence; and (ii) clinically, to evaluate the diagnostic performance of this assay in a pilot case-control study using 2 mL of plasma samples from an EO-CRC cohort.

### Reference Materials

Synthetic double-stranded gBlocks for *SEPT9*, *KCNQ5*, and *C9orf50* (IDT, Belgium) served as positive controls for the PCR systems, each spanning the respective DMR target regions (Table S1). Random sequences were each added at the 3′- and 5′-ends of the gBlocks to mitigate potential degradation by DNases. To simulate complex fragment mixtures found in plasma cfDNA eluates, we employed two types of cell culture-derived cfDNA surrogates. The healthy human wild-type control cfDNA surrogate (WT_cfDNA) was purchased as sheared DNA derived from an Ashkenazim son cell culture (SensID, Germany). For the CRC-derived ctDNA surrogate (HCT116_ctDNA), we extracted mono- and oligonucleosomes from cultured HCT116 cells using the Nucleosome Preparation Kit (Active motif, USA). The manufacturer’s instructions were followed for 15 million cells with an optimized 1 h lysis and final 15 min of incubation with an enzymatic shearing cocktail for effective digestion of linker DNA between nucleosomes. DNA cleanup was performed according to the manufacturer’s instructions, and further purification was done using QIAquick PCR Purification Kit (Qiagen, Germany). Size distribution was determined using the Fragment Analyzer (Agilent, USA) (Figure S1). All of the reference materials were stored at −20 °C until further use. Both WT_cfDNA and HCT116_ctDNA reference materials were confirmed in their methylation status of target regions for *SEPT9*, *KCNQ5,* and *C9orf50* using enzymatic methyl sequencing (CeGaT, Germany). The methylation rate of HCT116_ctDNA for the three targets was confirmed to be over 95%.

### Sample Population and DNA Extraction

For this study, plasma samples were collected from Rostock University Medical Center and University Medical Center Schleswig Holstein in Lübeck, aliquoted with volumes of at least 1.95 mL, and stored at −80 °C. Although no uniform plasma sample quality testing was performed across both sites, the Biobank Rostock and the Interdisciplinary Center for Biobanking-Lübeck (ICB-L) adhered to established standard operating procedures to maintain consistent biospecimen quality for this study. The CRC case population consisted of 32 EO-CRC patients across stages 0–IV who had been diagnosed before age 50 and were either primary, hereditary, or relapsed cases. The control population included 29 age- and gender-matched non-CRC controls, with and without gastrointestinal abnormalities. DNA extraction from plasma was performed using the QIAamp circulating nucleic acid kit (Qiagen, Germany) according to the manufacturer’s instructions. Purified cfDNA was eluted in 75 μL in LoBind tubes (Eppendorf SE, Germany), quantified using the Qubit dsDNA High Sensitivity Assay Kit (ThermoFisher Scientific, USA), and stored at −20 °C until further use (<1 month) or at 4 °C when used within 24 h.

### Primer and Probe Design and In Silico Testing

Primers were designed for the selected target sequences using the publicly available Primer-BLAST tool.^45^ The design criteria were adapted from Apte and Daniel,^46^ with specific settings detailed in Table S2. Each generated primer sequence was then blasted against RefSeq curated genomes of Homo sapiens, Arabidopsis thaliana, and *lambda* phage to ensure specificity by excluding primer pairs that generated unintended targets under 300 base pairs in silico. The design and optimization of mediator probes for the hydrolysis-based mediator probe PCR (MP PCR) technology were performed using AssayManager (GNWI mbH, Germany). The assay design and fluorophore selection were guided by the methodologies previously described.^47,48^ All primer, mediator probe, and universal reporter oligonucleotides (sequences provided in Table S3) were synthesized by biomers.net (Germany).

### cfMeDIP Protocol

DNA immunoprecipitation assays were performed using the MagMeDIP Kit (Diagenode, Belgium) following the manufacturer’s instructions with adjustments for cfDNA as previously described.^20^ A detailed protocol of the cfMeDIP procedure, including minor modifications from the original, is provided in Figure S2. Briefly, input DNA, when below 100 ng, was topped up with filler *lambda* DNA, which consisted of a mixture of unmethylated and in vitro methylated *lambda* phage amplicons of varying CpG densities (details in Table S4). The sample DNA/filler DNA mixture was combined with 0.5 ng of control methylated and 0.5 ng of control unmethylated A. thaliana DNA provided in the kit, along with the respective buffers. The mixture was first heated to 95 °C for 10 min and then rapidly cooled in an ice water bath for 10 min. Each sample was divided into two 0.2 mL PCR tubes: one for the input control (IC containing 7.5 μL) and the other for the sample to be subjected to immunoprecipitation (IP containing 75 μL). The included 5 mC monoclonal antibody 33D3 (C15200081) from the MagMeDIP Kit was added at 176 ng per IP reaction, followed by the addition of magnetic beads, which were washed according to the manufacturer’s instructions. Both IP and IC samples were incubated at 4 °C for 17 h before being purified using the IPure Kit v2 (Diagenode, Belgium) and eluted twice in 25 μL of buffer C to yield a final eluate of 50 μL. The success of the immunoprecipitation was validated by qPCR to evaluate recovery and specificity of the spiked-in methylated and unmethylated A. thaliana DNA. Recoveries from Ct values were calculated as

$$recovery=2^{\left({Ct}_{IC,10\%}-3.32-{Ct}_{IP}\right)}\times 100\%$$

Samples were only further analyzed if the percentage recovery of methylated spiked-in DNA exceeded 20% and was below 1% for unmethylated spiked-in DNA, respectively (relative to IC, adjusted for IC being 10% of the initial sample). Additionally, the reaction’s specificity had to reach over 99%, calculated as

$$specificity=\left(1-\frac{\%\;recovery\;of\;unmethylated\;spike-in\;DNA}{\%\;recovery\;of\;methylated\;spike-in\;DNA}\right)\times 100\%$$

### PCR Conditions and Instruments for Evaluation of PCR Systems

All reagents, except for template DNA, were prepared in an isolated pre-PCR room to avoid contamination. The qPCR reactions performed for primer evaluation were prepared using Perfecta multiplex qPCR ToughMix (Quanta Biosciences, USA), 0.5 μL of EvaGreen Dye (Biotium, USA), 400 nM of each forward and reverse primer, 1 μL of DNA template, and topped up with nuclease-free water to a final volume of 10 μL (Table S5). The templates were amplified on QuantStudio 5S (ThermoFisher Scientific, USA) with the following settings: initial denaturation at 95 °C for 5 min, 45 cycles of (i) denaturation at 95 °C for 15 s and (ii) annealing/extension at 58 °C, 60 °C, and 62 °C for 60 s. A subsequent melting curve analysis was conducted at 95 °C for 15 s, 60 °C for 60 s, followed by 95 °C for 15 s (Table S6). Synthetic DNA (gBlocks) served as the positive control, and nuclease-free water served as the negative control.

The dPCRs were set up in a final volume of 25 μL including 9.5 μL of DNA template, 2.5 μL of Buffer A, and 1 μL of Buffer B from the Naica multiplex PCR MIX 10× kit (Stilla Technologies, France), 500 nM of each forward and reverse primers, 400 nM of each universal reporter, and 1.2 μM of each mediator probe, topped up with nuclease-free water. The cycling protocol is detailed in Table S7. Assays were loaded onto Sapphire chips, processed on a Naica Geode cycler for droplet generation, and scanned in a Naica Prism6 scanner with the following default absorption wavelength exposure times. Positive (HCT116_ctDNA) and negative controls (nuclease-free water) were run in at least one replicate per Mastermix.

### Data Analysis

The dPCR threshold for each color channel was manually set to exclude the droplet population in the negative control; a 95% confidence interval was calculated by the Crystal Miner software. Methylation ratios were calculated as follows

$$methylation\;ratio=\frac{target\;concentration\;in\;the\;IP\;sample}{target\;concentration\;in\;the\;IC\;sample\times 10}\times 100\%$$

For each individual in our study cohort, the mean methylation ratio from duplicate tests was used for the determination of assay performance. The outcome variable (EO-CRC/non-CRC) was handled as binary, while the methylation ratio was treated as continuous. The cutoff values were determined using the Youden index^49^ unless otherwise specified. All statistical analyses and sensitivity and specificity calculations were done with R version 4.4.1. The ggplot2 package was used for creating boxplots and bar graphs, and the pROC package was used for generating ROC curves. Differences between median values were analyzed using the Mann–Whitney *U* test, with *p* ≤ 0.05 considered as statistically significant.

## Results and Discussion

### Selection of CRC Methylation Markers

In order to select CRC-specific methylation markers in cfDNA, suitable for the transfer to a bisulfite-free cfMeDIP-dPCR panel, available publications were evaluated based on three criteria: (i) markers were identified through bisulfite-based methods, providing base-pair resolution of methylation status at CpG-sites; (ii) the publication provides detailed information about the exact genomic location of the PCR amplicon; and (iii) the clinical validation was specifically conducted on blood plasma samples from CRC patients to demonstrate clinical significance in a liquid biopsy setting.

Two publications meeting these criteria were selected as the basis for our cfMeDIP-dPCR panel design: Ma et al. developed a bisulfite-based dPCR assay for quantification of methylated and unmethylated *SEPT9* from plasma in 103 CRC patients across the stages I–IV and 32 non-CRC controls.^50^ Using this assay, the authors determined methylation abundance, defined as the ratio of methylated copies per total number of copies, achieving a sensitivity of 74% at a specificity of 50%. Inclusion of the *SEPT9* target in our panel was of particular interest since it is the first blood-based methylation marker approved by the Food and Drug Administration (FDA) for CRC screening. Jensen et al. identified CRC-specific DMRs in three genes, *KCNQ5*, *C9orf50*, and *CLIP4*, and established a bisulfite-based dPCR triplex assay for CRC detection, termed TriMeth.^30^ Their comprehensive biomarker discovery process involved screening a data set of 5820 DNA methylation profiles from various tumors and blood cell populations utilizing InfiniumMethylation450 BeadChips. This was followed by validation of the triplex dPCR assay on plasma samples from a total of 256 CRC patients across stages I–IV and 178 non-CRC controls with a sensitivity of 85% at a 99% specificity.

### Establishment and Technical Evaluation of cfMeDIP-Compatible dPCR Assays for CRC

It is important to note that bisulfite treatment converts unmethylated cytosine to uracil, which is ultimately replaced by thymine in the newly synthesized DNA strand during the PCR. Hence, in this work, primer and probe design had to be done anew since cfMeDIP-dPCR targets original, bisulfite-untreated sequences. This resulted in primer pairs with an average increase in the GC content of 20% in comparison to published primer pairs from Jensen et al. and Ma et al.,^30,50^ rendering the PCR primer design as the main challenge. The PCR systems developed in this study were the result of a stepwise selection process (Figure 1a and Table S8), starting with an in silico design of primer pairs for all markers (*KCNQ5*, *SEPT9*, *C9orf50*, and *CLIP4*) using Primer-BLAST. We introduced stringency through two main criteria: First, the amplicon size was limited to 119 bp, as longer amplicons may drastically reduce the number of cfDNA fragments (median size of 167 bp) from being suitable PCR templates.^51^ Second, any systems generating unintended targets of 300 bp or below were excluded when blasted against reference genome sequences from H. sapiens, A. thaliana (spike-in control for cfMeDIP), and *lambda* phage (filler DNA used during cfMeDIP). This ultimately led to the design of six primer pairs for *SEPT9*, nine for *KCNQ5*, and three for *C9orf50* (Table S9). *CLIP4* was excluded from the panel since the particularly high GC content (81.6% for the published sequence—see Table S10) and the highly repetitive nature of its sequence made it unfeasible for us to design specific primers around the published target region without amplifying off-target sequences in silico.

**Figure 1:**
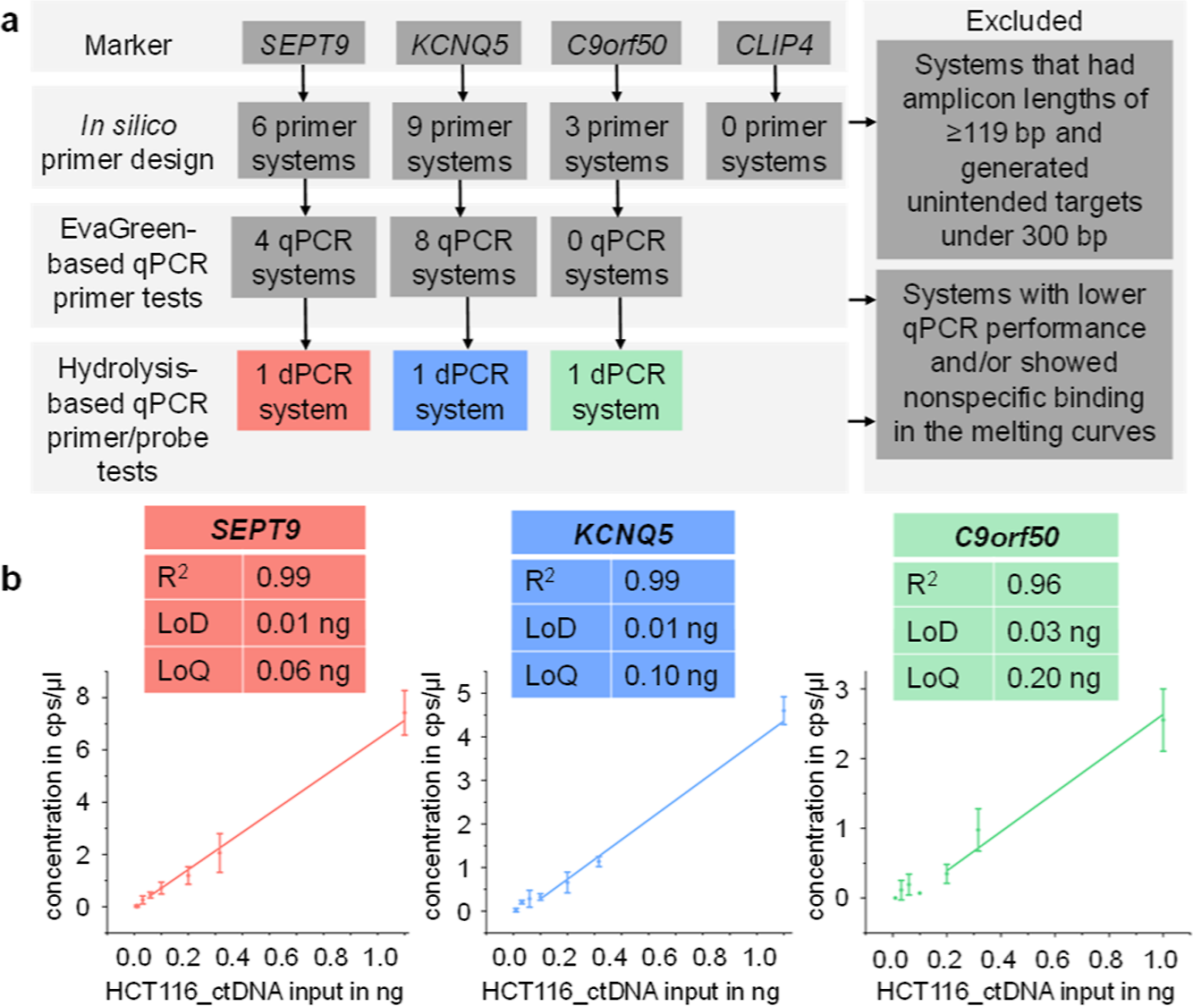
(a) Stepwise testing strategy for the design of a multiplexed dPCR panel, starting with four published methylation markers (*SEPT9*, *KCNQ5*, *C9orf50*, and *CLIP4*). (b) The three final marker candidates (*SEPT9*, *KCNQ5*, and *C9orf50*) were combined into a triplex dPCR system for LoD and LoQ testing. Each concentration in the 7-point adjusted semilogarithmic dilution series using the CRC cell line ctDNA surrogate (HCT116_ctDNA) was analyzed in a technical triplicate. Data are presented as mean ± SD. LoD: limit of detection, LoQ: limit of quantification.

The in vitro testing began with evaluating primer pairs using an EvaGreen qPCR assay, in which specificity was assessed through melting curve analysis. EvaGreen-based qPCR testing was performed in four levels, each testing on different DNA templates (Table S8). Briefly, qPCR systems were tested on synthetic DNA containing the DMR, referred to as gBlocks (level 1), ctDNA surrogate modeling CRC (level 2), which was derived from enzymatically fragmented mononucleosomal DNA (mnDNA) from HCT116 cells (further referred to as HCT116_ctDNA), cfDNA surrogate modeling non-CRC (level 3), which was derived from an Ashkenazim son cell culture, further referred to as WT_cfDNA, and last on *lambda* filler DNA and A. thaliana spike-in controls (level 4), representing the DNA background present in the cfMeDIP protocol. During testing by EvaGreen qPCR, all three *C9orf50* primer pairs exhibited more than one peak in the melting curves, suggesting the amplification of additional, nonspecific PCR products (Table S8 and Figure S3), while most primer pairs for the detection of *SEPT9* (4 out of 6) and *KCNQ5* (8 out of 9) displayed single, specific peaks in their melting curves.

To enable multiplexed detection, the hydrolysis-based mediator probe PCR (MP PCR) technology was subsequently employed, described by our group before as a sensitive and specific method for quantification of ctDNA.^52,53^ The inclusion of mediator probes improved system specificity as amplification occurs only when both the primer pair and the probe successfully bind to the template. Upon cleavage during amplification, the mediator probe (MP) oligonucleotide binds to a universal reporter (UR), which generates a fluorescent signal. This decouples target DNA recognition from signal generation, allowing the use of preoptimized URs with high fluorescence signal-to-noise ratios, independent of the target sequence.^47^ MP PCR added specificity, enabling the reevaluation of the three previously excluded *C9orf50* primer pairs. Of these, one system resulted in an amplification curve and was incorporated into the final triplex panel.

The technical sensitivity of our triplex dPCR assay was evaluated using a 7-point adjusted semilogarithmic dilution series. HCT116_ctDNA inputs of 1, 0.316, 0.2, 0.1, 0.06, 0.0316, and 0.01 ng were spiked into a constant background of a 1 ng filler DNA mixture. Each concentration was analyzed in technical triplicate. The linear range was defined as the concentration range that produced at least three positive dPCR droplets for all replicates and exhibited a coefficient of variation (CV) of less than 50%. *R* ^2^ was determined from the linear regression fit through this linear range. The limit of quantification (LoQ) was determined as the lowest concentration within this linear range, whereas the limit of detection (LoD) was determined as the concentration at which at least one of the technical replicates had more than 3 positive droplets. The LoD and LoQ were 0.01 and 0.06 ng for *SEPT9*, 0.01 and 0.1 ng for *KCNQ5*, and 0.03 and 0.2 ng for *C9orf50* (Figure 1b and Table S11). Both *KCNQ5* and *SEPT9* exhibited good linearity, each with an *R* ^2^ value of 0.99, whereas *C9orf50* displayed a lower linearity with an *R* ^2^ of 0.96. These findings highlight the challenges of adapting bisulfite-based assays to cfMeDIP-dPCR, particularly regarding primer design for GC-rich regions. The higher GC content of the cfMeDIP-compatible primer sets led to specificity issues, as seen with *C9orf50*, and ultimately prevented the inclusion of *CLIP4* in the final panel. Additionally, analytical performance metrics, including *R* ^2^ values and detection limits, indicated that markers with a lower GC content such as *SEPT9* and *KCNQ5* performed more reliably. Since high GC content can negatively impact both primer design and overall assay performance, selecting target regions downstream of the promoter and transcription start site, where GC content is typically lower,^54^ should be considered. However, since this study focused on the feasibility of transferring published target sequences, alternative regions were not explored.

### Technical Evaluation of cfMeDIP Coupled with dPCR

We assessed the compatibility of cfMeDIP sample preparation (Figure 2a) with our dPCR triplex assay using varying input amounts of cfDNA surrogates. A dilution series of HCT116_ctDNA (10 ng, 5 ng, 1 ng) was subjected to cfMeDIP sample preparation, according to the previously published cfMeDIP protocol^20^ with minor changes detailed in Figure S2. Each concentration was processed in two cfMeDIP replicates. For each cfMeDIP replicate, two sample fractions were generated: the input control (IC), representing the total fraction before immunoprecipitation, and the immunoprecipitation (IP) sample, representing the methylated fraction. Both the IC and IP samples were analyzed in four technical dPCR replicates each, enabling individual assessment of technical variabilities of the cfMeDIP sample preparation and of the dPCR analysis. The methylation ratio for each marker was calculated as the concentration of positive droplets in the IP sample divided by the volume-corrected concentration of positive droplets in the corresponding IC sample (Figure 2a). Consequently, four methylation ratios were obtained per cfMeDIP replicate for each marker (Figure 2b).

**Figure 2:**
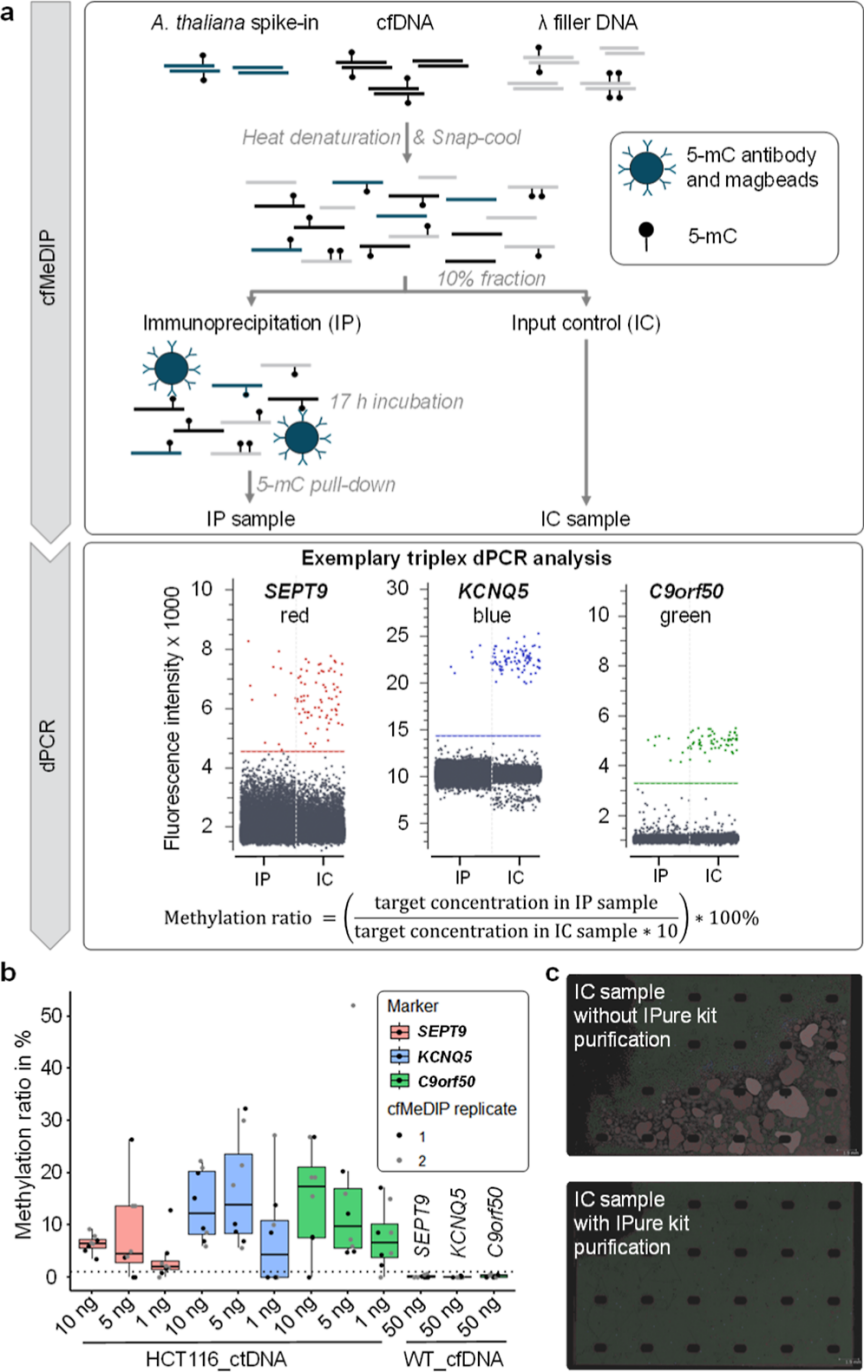
(a) cfMeDIP-dPCR workflow with the 2D dot-plot of an exemplary triplex dPCR analysis for a case with low methylation ratio. (b) Technical evaluation of the cfMeDIP-dPCR triplex assay. dPCR replicates from cfMeDIP replicate 1 are marked as black dots and replicate 2 as gray. Methylation ratios above 1% were considered detectable (dotted threshold line). Boxplots display the interquartile range (box), median (line), and range (whiskers). (c) Exemplary images of IC samples in droplet chips without (top) and with (bottom) IPure purification. Merged droplets appear in red (marked by the software). cfMeDIP: cell-free methylated DNA immunoprecipitation, HCT116_ctDNA: CRC ctDNA surrogate from HCT116 cell culture, and WT_cfDNA: wild-type cfDNA surrogate from Ashkenazim son cell culture.

Both IC and IP samples were purified using the IPure kit to remove residual buffers. This purification step was crucial, as the presence of cfMeDIP buffers in the IC samples caused droplet merging during dPCR analysis (Figure 2c and Table S11). To evaluate specificity, 50 ng of WT_cfDNA was subjected to cfMeDIP sample preparation. This amount covers the 95th percentile of DNA input amount (range 2.76–475.94 ng, median 13.94 ng) from the clinical cohort tested in this study. As expected, none of the markers exhibited methylation ratios above 1% with 50 ng of WT_cfDNA, confirming the specificity of the assay. Consequently, a threshold of 1% was defined for detectable methylation ratios.

The median methylation ratios for all markers exceeded this threshold across all tested input concentrations, with a decrease observed as the input concentration was reduced Notably, while the ranges in the methylation ratio within each cfMeDIP replicate exhibited substantial variability, with ranges spanning up to 47%, the ranges in methylation ratios between both cfMeDIP replicates were largely overlapping. This suggests that the technical variability of the dPCR replicates is of greater concern than that of the cfMeDIP replicates. The variability primarily arises from the calculation method, which depends on the ratio of positive droplet concentrations between the IP and IC samples. Given that the number of positive droplets for both samples is often in the single-digit range, even minor fluctuations in either measurement disproportionately affect the final methylation ratio. To account for this, we implemented two measures for analysis of the clinical cohort: (i) concentrations outside of the 95% confidence interval, as calculated from the dPCR readout software, were considered negative to exclude uncertain droplet counts and (ii) each IC and IP sample was analyzed in dPCR duplicate runs. This approach provides two methylation ratios per sample, ensuring a more accurate diagnosis while maintaining a practical number of replicates for clinical workflows.

### Clinical Evaluation of the cfMeDIP-dPCR Triplex Assay on Plasma Samples to Identify EO-CRC Patients

To evaluate the diagnostic performance of the cfMeDIP-dPCR triplex assay in plasma, we tested a cohort of 32 EO-CRC patients and 29 age- and gender-matched non-CRC controls (Table S13) from the German national OUTLIVE-CRC study. We limited the plasma volume per individual to 2 mL (range 1.95–2.30 mL, median 1.95 mL). For each individual, a fixed eluate input volume of 53 μL from cfDNA extraction was processed through the cfMeDIP protocol. Since both the IP and IC samples were each analyzed in duplicate dPCR runs, the actual plasma volume contributing to the final analysis corresponds to just 537 μL per individual (for calculations, see Table S14).

The mean methylation ratios were used for determining cutoff values and calculating sensitivities and specificities for both individual markers and marker combinations. The median methylation ratio for each marker was significantly higher in EO-CRC patients compared to non-CRC individuals (*p* ≤ 0.001, Figure 3a), confirming their high specificity for CRC. Receiver operating characteristic (ROC) curves and corresponding area under the curve (AUC) values demonstrated discriminatory power between EO-CRC and non-CRC: AUC = 0.85 (*SEPT9*), 0.92 (*KCNQ5*), and 0.86 (*C9orf50*) (Figure 3b).

**Figure 3:**
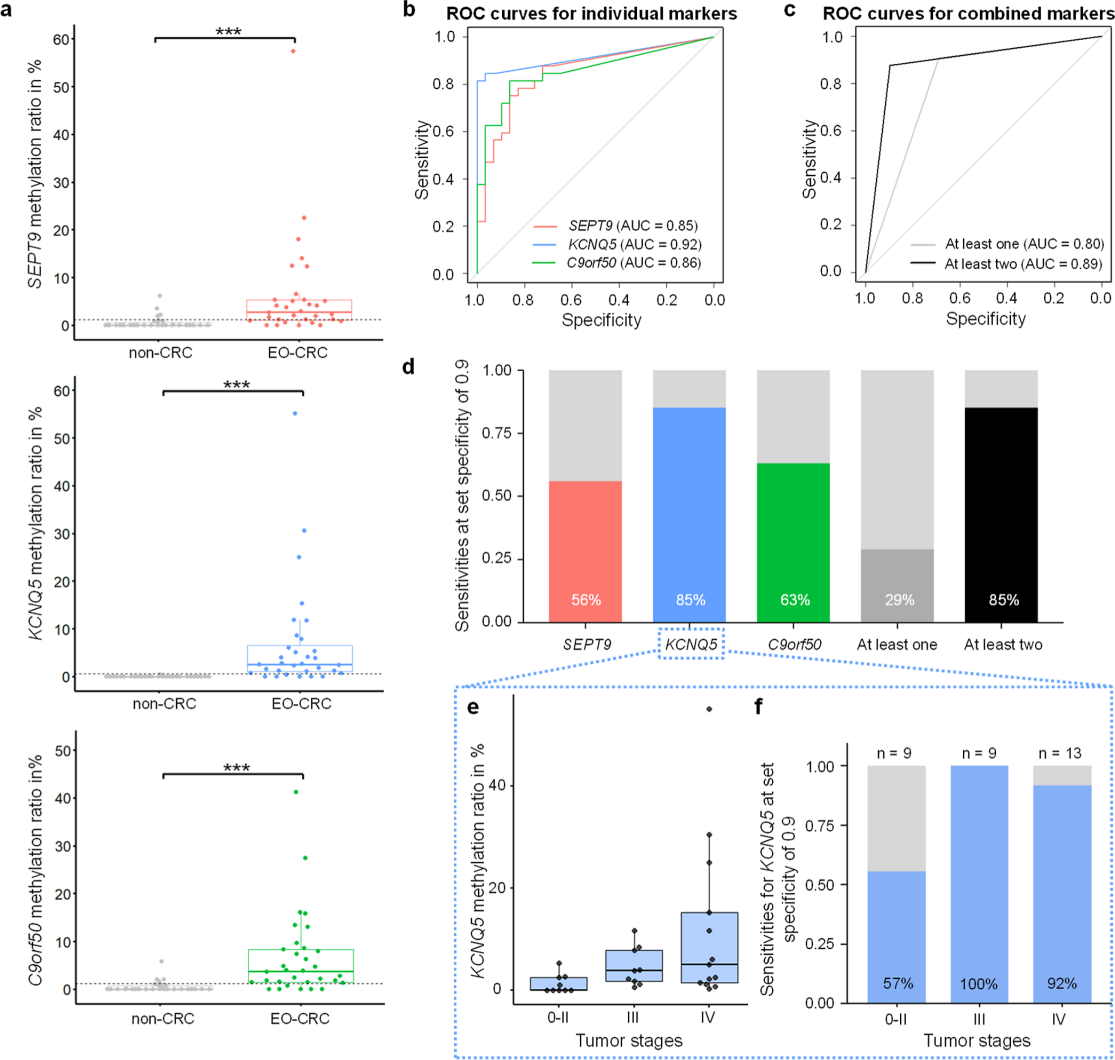
Detection of *SEPT9*, *KCNQ5*, and *C9orf50* markers in plasma. (a) Boxplots of mean methylation ratios of individual markers in 32 EO-CRC patients and 29 non-CRC individuals. The gray dotted line shows the diagnostic cutoff value for each marker, determined using the Youden index (0.23% for *SEPT9*, 0.59% for *KCNQ5*, and 1.29% for *C9orf50*). Mann–Whitney *U* test to compare between two groups; ***p ≤ 0.001. (b) ROC curves for individual markers *SEPT9* (red), *KCNQ5* (blue), and *C9orf50* (green) with their respective AUC values. (c) ROC curves for combined approaches using the “At least one” and “At least two” approach. (d) Sensitivities for all individual markers, as well as combined markers, with a set specificity of 0.9. (e) *KCNQ5* methylation ratios across grouped tumor stages 0–II, stage III, and stage IV (patient with missing information about tumor stage excluded). (f) Sensitivities of *KCNQ5* at a set specificity of 0.9 across grouped tumor stages 0-II, stage III, and stage IV (patient with missing information about tumor stage excluded). EO-CRC: early-onset CRC, ROC: receiver operating characteristic, and AUC: area under the curve.

To evaluate whether combining markers could improve diagnostic performance, we tested two combinatorial approaches as suggested by Jensen et al.:^30^ the “At least one” approach, where a sample was considered positive if at least one marker exceeded the cutoff, and the “At least two” approach, requiring two markers to exceed the cutoff. To implement these approaches, cutoff values for each marker were determined using the Youden index.^49^ ROC curves were then generated for the combined approaches, yielding AUC values of 0.80 (“At least one”) and 0.89 (“At least two”) (Figure 3c). However, these combined approaches showed no improvement over the individual marker *KCNQ5*.

To enable direct comparison of individual and combined marker performance, sensitivities were calculated at a fixed specificity of 90% as this threshold aligns with the guidelines from the US Centers for Medicare and Medicaid Services (CMS) for blood-based CRC assays (decision memo: CAG-00454N).^55^ At this specificity, *KCNQ5* as a single marker and the “At least two” approach both achieved the highest sensitivity (85%) and the “At least one” approach showed the lowest sensitivity (29%). Given the superior performance of *KCNQ5*, compared to other single markers in this study, we further investigated whether methylation ratios and sensitivities of *KCNQ5* correlated with the tumor stage. Due to the limited number of samples from lower tumor stages, stages 0–II were grouped for analysis (*n* = 9). *KCNQ5* methylation ratios significantly correlated with the tumor stage (Spearman’s rank correlation, *p* ≤ 0.05) (Figure 3e), and the marker demonstrated particularly high sensitivity at tumor stage III (100%, *n* = 9) and stage IV (92%, *n* = 13) (Figure 3f).

While *KCNQ5* reached comparable clinical diagnostic performance (85% sensitivity at 90% specificity) to the single marker used in the TriMeth assay (83% sensitivity at 95% specificity),^30^ *C9orf50* had significantly lower clinical diagnostic performance (63% sensitivity at 90% specificity) compared to the single marker from the TriMeth assay (76% sensitivity at 91% specificity). Notably, the TriMeth assay effectively used 11,520 μL of plasma, whereas our study achieved comparable sensitivity using only 537 μL of plasma equivalent input, representing a 21-fold reduction in the required sample volume (Table S14). It remains unclear whether different marker performances between our study and others are due to differences in assay performance, differences in analyzed plasma volumes, or differences in the cohorts, e.g., in the mean age of subjects. For *SEPT9*, a marker widely referenced in the context of CRC detection, the diagnostic performance was the lowest among our panel (56% sensitivity at 90% specificity). Compared to the original publication from which we derived the DMR for *SEPT9* (74% sensitivity at 50% specificity), our study demonstrated higher sensitivity at the same specificity level (91% sensitivity at 50% specificity). In broader comparison, a meta-analysis of published case-control studies using *SEPT9* as a single marker reported a pooled sensitivity of 74% and a specificity of 84%,^56^ while Loomans-Kropp et al. recently reported a 91% sensitivity at an 89% specificity in a cohort of 27 EO-CRC and 87 non-CRC controls.^57^ These findings suggest that exploring alternative DMRs for *SEPT9* could further improve the cfMeDIP-dPCR assay performance.

## Conclusions

This study introduces multiplexed cfMeDIP-dPCR as an innovative and clinically feasible assay for cancer detection through epigenetic signatures in liquid biopsies. Demonstrated in a pilot study with EO-CRC cases and non-CRC controls, this assay addresses a critical diagnostic need with the advantage of requiring over 20 times less plasma than a sensitivity-matched bisulfite-based assay. The strong correlation between methylation ratio and tumor stage, as shown for the most promising marker in our panel, further underscores its clinical relevance, enabling not only cancer detection but potentially also treatment monitoring. These findings pave the way for more accessible and patient-friendly diagnostic tools, enhancing early detection efforts for EO-CRC and other diseases with epigenetic markers. Future studies with larger, more diverse cohorts are essential to validate these findings and optimize the assay performance.

## Supplementary Material



## Notes

Fragment size analysis of HCT116_ctDNA; detailed description of the adapted cfMeDIP protocol; experimental analysis of buffer effects on droplet formation in dPCR; calculations for effectively tested plasma volumes; list of members of the OUTLIVE-CRC consortium; sequences of templates, primers, and targets; parameters for primer design; information about the filler DNA fragments; composition and protocols of the PCRs; experiments for primer testing; melting curves; results of the LoD and LoB measurements; as well as patient and control characteristics (PDF)
