# Supplementary material for: Coupling Immunoprecipitation with Multiplexed Digital PCR for Cell-Free DNA Methylation Detection in Small Plasma Volumes of Early-Onset Colorectal Cancer
Source: Anal Chem. 2025 May 17;97(21):11259–68. doi: 10.1021/acs.analchem.5c01361 (PMC12138876; doi:10.1021/acs.analchem.5c01361)
Supplement: Supplementary file 1 [file ac5c01361_si_001.pdf]

Supporting Information

Coupling immunoprecipitation with multiplexed digital PCR for cell-free DNA methylation detection in small plasma volumes of early-onset colorectal cancer

Truong T. Truong[a*], Klara Mikloska[b], Judith Sum[b], Martina Oberländer[c], Nikolas von Bubnoff[d,e], Lea Christiansen[f], Sebastian Tornow[f], Stefanie Derer[f], Florian Janke[g,h,i], Holger Sültmann[g,h,i], Sebastian Zeissig[j], Michael Linnebacher[k], Clemens Schafmayer[l], Michael Lehnert[a], Tobias Hutzenlaub[a,b,e], Nils Paust[a,b], and Peter Juelg[a,b,e]

[a]Hahn-Schickard, 79110 Freiburg, Germany; [b]Laboratory for MEMS Applications, IMTEK – Department of Microsystems Engineering, University of Freiburg, 79110 Freiburg, Germany; [c]Interdisciplinary Center for Biobanking-Lübeck (ICB-L), University Medical Center Schleswig-Holstein, 23562 Lübeck, Germany; [d]Department of Hematology and Oncology, University Medical Center Schleswig-Holstein, 23538 Lübeck, Germany; [e]European Liquid Biopsy Society (ELBS), 20246 Hamburg, Germany; [f]Institute of Nutritional Medicine, University Medical Center Schleswig-Holstein, 23562 Lübeck, Germany; [g]Division of Cancer Genome Research, German Cancer Research Center (DKFZ), 69120 Heidelberg, Germany; [h]Translational Lung Research Center (TLRC), Member of the German Center for Lung Research (DZL), 69120 Heidelberg, Germany; [i]National Center for Tumor Diseases (NCT), 69120 Heidelberg, Germany; [j]Department of Internal Medicine A, University Medical Center Greifswald, 17475 Greifswald, Germany; [k]Biobank Rostock, Clinic of Surgery, Rostock University Medical Center, 18057 Rostock, Germany; [l]Clinic of Surgery, Rostock University Medical Center, 18057 Rostock, Germany

*Email: Tu.Truong@imtek.uni-freiburg.de

Table of contents

Table S1: gBlock sequences used as positive controls S2
Fig. S1: Fragment size analysis of HCT116_ctDNA S3
Table S2: Parameters for Primer-BLAST S4
Table S3: Oligonucleotide sequences S5
Fig. S2: Adapted cfMeDIP protocol S6
Table S4: Filler DNA fragments S10
Table S5: qPCR composition and level testing strategy S11
Table S6: qPCR cycling conditions S12
Table S7: dPCR cycling conditions S13
Table S8: Results of level testing S14
Table S9: Selected primer pairs for each target S15
Table S10: Target sequences of selected targets used for primer design with Primer-BLAST S17
Fig. S3: Exemplary melting curve of C9orf50 S18
Table S11: Results of LoD and LoQ experiments S19
Table S12: Experimental analysis of buffer effects on droplet formation in dPCR S21
Table S13: Patient and control characteristics. S23
Table S14: Effective plasma volume calculation S24
Table S15: Members of OUTLIVE-CRC consortium S25
References S26

Table S1: Sequences of ordered gBlocks (Integrated DNA Technologies) used as positive control in PCR experiments. Each gBlock contained the target sequence of the corresponding DMR (shown in black). The two random sequences at the 5' and 3' end of the target sequence are illustrated in blue and orange. DMR: Differentially methylated region.

Name	Target or DMR	Size / bp	Sequence (5'-…- 3')
KCNQ5_control	*KCNQ5*	176	ATTTGACATGACTTATGAACTGTAGTCTGGGGAAGCCGCTCTCTTACACGAGTAGCCAGAGCTGCCGGCGCAACGTCAAGTACCGGCGG GTGCAGAACTACCTGTACAACGTGCTGGAGAGACCCCGCGGCTGGGCGTTCATCTACCACCTGTACTCATGTTCAATCAGTGTACTG
SEPT9_control	*SEPT9*	178	ATTTGACATGACTTATGAACTGTAGTTTGTTTGGCTGCCCAAATACAGCCTCCTGCAGAAGGACCCTGCGCCCGGGGAAGGGGAGGAAT CTCTTCCCCTCTGGGCGCCCGCCCTCCTCGCCATGGCCCGGCCTCCACATCCGCCCACATCTCTGTACTCATGTTCAATCAGTGTACTG
C9orf50_control	*C9orf50*	142	ATTTGACATGACTTATGAACTGTAGTCAAGAAGTCGGGGTCCTCCCTGGCCACGCGCCTCCGGGGGCGCTCGCGCTCTCCAGGCCCTGGC TGCCTGGGCGCCGATTCCCGGGACGCTGTACTCATGTTCAATCAGTGTACTG

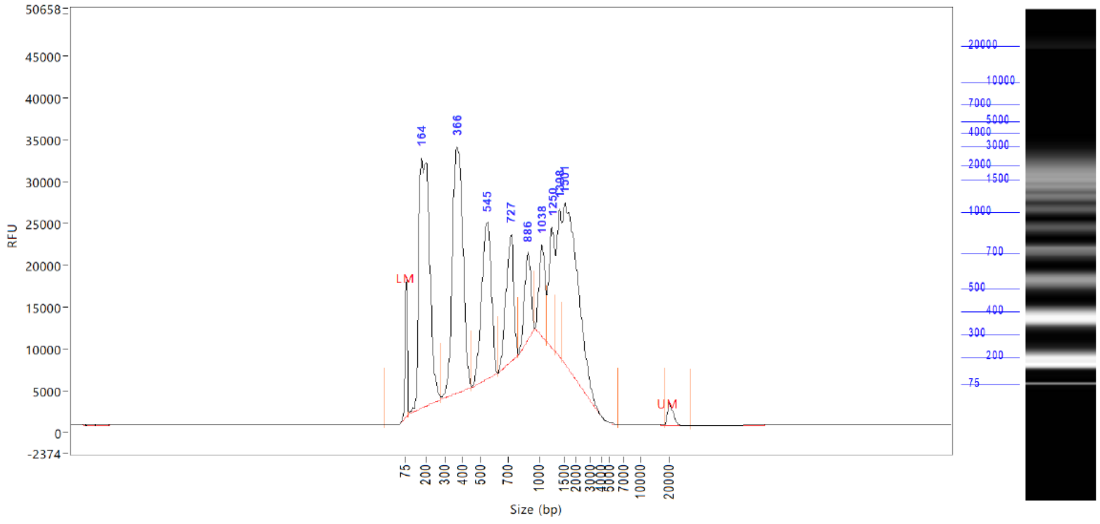

Concentration (in ng/µl) and relative concentration (in %) of each detected RFU peak obtained from analyzing HCT116_ctDNA in automated electrophoresis run on Fragment Analyzer (Agilent, Santa Clara, CA, USA). RFU: Relative fluorescence units.

RFU peak	Size / bp	Concentration / (ng/µl)	Relative concentration / %
1	164	63	24.9
2	366	55	21.6
3	545	30	11.9
4	727	18	7.1
5	886	10	4.0
6	1038	10	3.9
7	1250	12	4.7
8	1398	14	5.4
9	1501	42	16.5

Fig. S1: Fragment size analysis of HCT116_ctDNA (obtained from CRC originated HCT116 cell culture) by Fragment Analyzer (Agilent, USA).

Table S2: Used parameters for primer design in tool Primer-BLAST. Parameters that are not listed in this table were adopted from Primer-BLAST default. Tm: Melting temperature. Bp: Base pairs. PCR: Polymerase chain reaction.

**Search Parameter in *Primer-BLAST***	**Value**
Min. PCR product size	45-60 bp
Max. PCR product size	110-119 bp
Min. T_m	57 °C
Optimal T_m	60 °C
Max. T_m	63 °C
Max. T_m difference	3 °C
Min total mismatches	2
Min 3' end mismatches	2
Defined 3' end region length	5
Mismatch threshold to ignore targets	6
Max target amplicon size	500 bp
Min. Primer size	16 bp
Optimal Primer size	20 bp
Max. Primer size	25 bp
Min. Primer GC content	20%
Max. Primer GC content	80%
Repeat filter	Automatic
Low complexity filter	Yes

Table S3: Sequences of the primers, mediator probes and universal reporter for dPCR triplex.

Name	Specification	Sequence (5'- 3')	5' modification	3' modification	internal
Fwd_Primer_KCNQ5	Primer	GGAAGCCGCTCTCTTACACG			
Rev_Primer_KCNQ5	Primer	TACAGGTAGTTCTGCACCCG			
Fwd_Primer_C9orf50	Primer	CAAGAAGTCGGGGTCCTCC			
Rev_Primer_C9orf50	Primer	GTCCCGGGAATCGGCG			
Fwd_Primer_SEPT9	Primer	TTGGCTGCCCAAATACAGC			
Rev_Primer_SEPT9	Primer	GAAGAGATTCCTCCCCTTCCCC			
**UR06_Green_C3_FAM**	Universal Reporter	attgcgggagatgagacccgcaa**8**tgTTCAGTGAGCCTACCTGC CTTC	BMN-Q-535	C3-Spacer	**8**=dTFA M
**KCNQ5_5 UR06_FAM MP_1**	Mediator Probe	GGTAGGCTCACTGAACAGTAGCCAGAGCTGCCGG CGCAAC			
**UR05_Yellow_C3**	Universal Reporter	GACCGGCTAAGACGCGCCGGT**7**TGTTGCACCTGGG ACATCGACTAT	BMN-Q-535	C3-Spacer	**7**=dC-Rhodamin 6G
**C9orf50_2&3_UR05_M P**	Mediator Probe	CGATGTCCCAGGTGCAACGCTCTCCAGGCCCTGGC TGCCTG			
**UR02_Red_C3**	Universal Reporter	gaccggccaagacgcgccggt**4**tgttcactgaccgaactggagca	BMN-Q-535	C3-Spacer	**4** = dC-Dy-636
**SEPT9_6_UR02_MP_1**	Mediator Probe	CAGTTCGGTCAGTGAACTCCTGCAGAAGGACCCT GCGCCC			

Beads preparation

1. Wash Magbeads (11µl per IP) **twice** with ice-cold MagBuffer A (1x, 27.5 µl)
2. After wasing the beads, resuspend beads in MagBuffer A (1x, 22 µl)
3. Keep beads on ice until further use

Sample preparation

4. DNA input preparation (filler DNA mix (50:50) was used to fill up the DNA input to 100 ng):

Component	Quantity/ volume
DNA input	Range 1 ng to 100 ng (up to 1µg is possible)<br>→ DNA input volume of cfDNA eluate (obtained from clinical samples) was set to **53 µl**
Filler DNA mix (ratio 50:50)	**Optional**: Up to 99 ng
dH_2O (nuclease-free)	Up to 57 µl
In total:	57 µl

5. cfMeDIP Mastermix preparation (for one sample):

Component	Quantity/ volume
MagBuffer A (5x)	27.5 µl
MagBuffer B (1x)	6.9 µl
**Methylated** spike-in control (*A. thaliana*)	0.6 µl
**Unmethylated** spike-in control (*A. thaliana*)	0.6 µl
dH_2O (nuclease-free)	2.3 µl

6. Add cfMeDIP Mastermix to DNA input:

Component	Quantity/ volume
cfMeDIP Mastermix	33 µl
DNA input	57 µl
In total:	90 µl

7. Incubate sample at 95 °C for 10 min (heated lid at 105 °C) to denature the double-stranded DNA
8. Snap-cool the single-stranded DNA afterwards by transferring the sample quickly into an ice-water slush
9. Incubate sample for 10 min on ice

10. From now on keep the sample on ice to minimize DNA re-annealing
11. Split sample into:
    - Input control (**IC**): 7.5 µl
    - Immunoprecipitation sample (**IP**): 75 µl
12. Keep IC on ice and store it at 4°C overnight
13. Continue with IP in immunoprecipitation part

Immunoprecipitation

14. Prepare antibody dilution:

Component	Quantity/ volume
Antibody	1.1 µg
Ice-cold dH_2O (nuclease-free)	Up to 5 µl
In total:	5 µl

15. Prepare antibody mix with antibody concentration of 0.0352 µg/µl:

Component	Quantity/ volume
Diluted antibody	0.8 µl
Ice-cold water	1.6 µl
MagBuffer A (5x)	0.6 µl
MagBuffer C (1x)	2 µl
In total:	5 µl

16. Add antibody mix (5 µl) to IP sample (in total: 176 ng of antibody is added)
17. Add washed Magbeads (20 µl) to IP sample
18. Incubate IP sample on rotator (10 rpm) for about 17 h at 4 °C to 6 °C for immunoprecipitation

Washing

19. Spin down IP sample and put it on ice-cold magnetic rack to magnetize
20. Discard supernatant
21. Wash Magbeads **three times** with ice-cold MagWash Buffer-1 (100 µl)

    → Invert to resuspend and spin on rotator for 5 min at 4 °C

    → Briefly spin, place samples in magnetic rack, magnetize and discard supernatant
22. Wash Magbeads **three times** with ice-cold MagWash Buffer-1 (100 µl)

→ Invert to resuspend and spin on rotator for 5 min at 4 °C

→ Briefly spin, place samples in magnetic rack, magnetize and discard supernatant

23. After last washing step, keep pellet of IP sample on ice

Elution with IPure V2 kit (Diagenode)

24. From now on treat IC and IP sample in parallel and work at room temperature (RT)
25. Prepare elution buffer (EB) (Important here: both buffers need to be at RT before mixing together):

Component	Quantity/ volume
Buffer A	230.8 µl
Buffer B	9.2 µl
In total:	240 µl

26. Add Elution buffer (EB) to samples:

→ IC sample: 92. 5 µl

→ IP sample: **2 x** 50 µl (elution from MagBeads is carried out in two steps of 50 µl)

27. Rotate all samples (IC and IP) for 15 min at 10 rpm and RT
28. After 15 min, briefly spin, magnetize and transfer the supernatant in new PCR tubes
29. Repeat the elution step a second time **only** for IP samples (add again 50 µl of EB, rotate 15 min at 10 rpm and RT, magnetize, transfer supernatant)
30. Add 2 µl of Carrier to each sample (IC and IP)
31. Add 100 µl of isopropanol (≥ 99.5 %) to each sample
32. Add 10 µl of magnetic beads to each sample (**Important** here: beads settle fast in stock tube, so mix thoroughly by pipetting before taking out the bead solution)
33. Rotate all samples for 10 min at 10 rpm and RT
34. Briefly spin, place samples in magnetic rack, magnetize and discard supernatant
35. Wash beads with 100 µl of wash buffer 1
36. Rotate the samples for 5 min at 10 rpm at RT
37. Briefly spin, place samples in magnetic rack, magnetize and discard supernatant
38. Wash beads with 100 µl of wash buffer 2
39. Rotate the samples for 5 min at 10 rpm at RT
40. Briefly spin, place samples in magnetic rack, magnetize and discard supernatant (**Important** here: Try to remove all liquid remaining in tube)

41. To remove any residuals of wash buffer 2, open the lid of all tubes and air-dry the samples in sterile workbench under laminar air flow for about 5 min
42. DNA elution is carried out in **two steps** (2 x 25 µl):
    → Add 25 µl of buffer C
    → Rotate all samples for 15 min at 10 rpm and at RT
    → Briefly spin, place samples in magnetic rack, magnetize
    → **Transfer supernatant** in new tube
    → Add again 25 µl of buffer C to the tube which contains magnetic bead pellet
    → Repeat elution one more time as described above
43. In the end, there should be 50 µl of eluate for each IC and IP sample in the newly prepared tubes
44. Eluate is stored short-term (< 24 hours) at 4°C or long-term at -20°C until further analysis in qPCR (quality control of antibody) or dPCR (evaluation of target recoveries)

Fig. S2: Detailed description of adapted cfMeDIP protocol. Volumes were adjusted to preparation of one cfMeDIP replicate, which results in an IC and an IP sample. All reagents used in the described protocol were obtained from two commercially available kits: the MagMeDIP Kit (Diagenode, Belgium) and the IPure Kit V2 (Diagenode, Belgium).

Table S4: Details of the filler DNA fragments used in cfMeDIP preparation, including their names, fragment sizes, methylation statuses, and the composition of the 50:50 ratio (methylated to unmethylated fragments) in the filler DNA mix. This mix was added to adjust the total DNA input to 100 ng. The six filler DNA fragments were prepared following the protocol described by Shen et al.(2019) [1].

Filler DNA fragment name	Fragment size / bp	Status for cfMeDIP	Composition of filler DNA mix (50:50 ratio) / in %
1CpG	196	Methylated	10
5CpG	269	Methylated	10
10CpG	359	Methylated	10
15CpG	461	Methylated	10
20LCpG	495	Methylated	10
20SCpG	274	Unmethylated	50

Table S5: (A) Composition of qPCR mix for evaluation of designed primers. Volume of added DNA template was the same throughout the experiments whereas the DNA concentration varied. (B) Final template concentrations in qPCR reactions in primer test experiment (Level 1 to 4).

(A)

Reagents	Final Concentration	Volume / µl
PerfeCTa® MultiPlex qPCR ToughMix®	1 x	2
EvaGreen dye	1 x	0.5
Primer forward<br>Primer reverse	400 nM<br>400 nM	0.4<br>0.4
DNA template	Variable	1
dH_2O	-	5.7

(B)

		**Final template concentration in qPCR reaction**
	**Level 1**<br>Template: gBlocks	10^3 cps/µl
	**Level 2**<br>Template: HCT116_ctDNA	66.6 ng
	**Level 3**<br>Template: WT_cfDNA	0.2 ng/µl
	**Level 4**<br>Template: *A. thaliana* spike-in control and and filler DNA mix	0.05 pg/µl (*A. thaliana*)<br>91.5 pg/µl (filler DNA mix)

Table S6: PCR cycling and melt curve conditions for EvaGreen qPCR assay targeting DMRs in CRC-markers on QuantStudio 5s (ThermoFisher Scientific, USA).

Step	Temperature / °C	Time / s	Cycle	Temperature ramp / (°C / s)
Initial denaturation	95	300	1 x	1.6
Denaturation	95	15	45 x	2
Annealing/ Extension	58/60/62	60		2
Melt curve	95	15	1 x	1.6
	60	60	1 x	1.6
	95	15	1 x	0.1

Table S7: PCR cycling conditions for dPCR assay targeting DMRs in CRC-markers on naica system for Crystal Digital PCR (Stilla Technologies, France).

Step	Temperature / °C	Time / s	Cycle
Initial denaturation	95	180	1 x
Denaturation	95	15	60 x
Annealing/ Extension	58	60	

Table S8: (A) Illustration of experiment test of primers. The ordered primer pairs were tested level-wise. Each level contained a different template. Goals of this experiment were to test if the primer designs work and to sort out nonspecific primer pairs. (B) Criteria for primer selection after each level. Main criterion concerning all levels was to select primers that are highly specific. Primers were considered as specific when one peak at characteristic melting temperature Tm was present in melting curve.

(A)

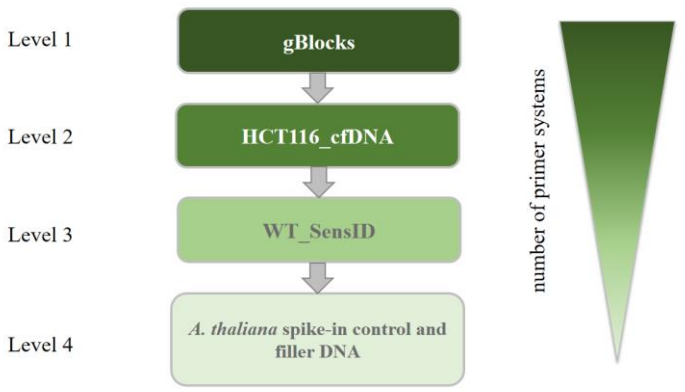

(B)

	Criteria for primer selection	Primer pairs (selected for next level)	Additional information
**Level 1**<br>Template: gBlocks	Select primers that show product peak at one T_m in melting curve to test functionality of designed primers and to identify characteristic T_m	*KCNQ5:* 9/9<br>*SEPT9:* 6/6<br>*C9orf50:* 3/3	Annealing temperatures: 58-60-62 °C
**Level 2**<br>Template: HCT116_ctDNA	Select max. **four** primer pairs for each DMR that generate shortest amplicon to facilitate target detection on cfDNA	*KCNQ5:* 4/9<br>*SEPT9:* 4/6<br>*C9orf50:* 0/3	Annealing temperature: 58 °C
**Level 3**<br>Template: WT_cfDNA	Select max. **two** primer pairs for each DMR that generate lowest Ct-value to determine more sensitive primer pairs under the applied qPCR conditions	*KCNQ5:* 2/9<br>*SEPT9:* 2/6<br>*C9orf50:* 0/3	Annealing temperature: 60 °C
**Level 4**<br>Template: *A. thaliana* spike-in control and and filler DNA mix	Select **one** primer pair for each DMR that facilitates specific mediator probe design	*KCNQ5:* 1/9<br>*SEPT9:* 1/6<br>*C9orf50:* 0/3	Annealing temperature: 60 °C

Table S9: Selected and ordered primer pairs for each DMR: Six for SEPT9, nine for KCNQ5, and three for C9orf50. Fwd: Forward. Rev: Reverse. Tm: Melting temperature.

Primer ID	Primer pair number	Target	Primer	Primer sequence (5'-…- 3')	Primer size / bp	Amplicon size / bp	T_m / °C	G/C content / %	Comments on *Primer-BLAST*
**P3084**	1	***KCNQ5***	fwd	GAAGCCGCTCTCTTACACG	19	73	58	58	no unintended targets under 500 bp
**P3085**			rev	CAGGTAGTTCTGCACCCGC	19		61	63	
**P3086**	2		fwd	GAAGCCGCTCTCTTACACGA	20	89	60	55	no unintended targets under 500 bp
**P3087**			rev	TCTCCAGCACGTTGTACAGG	20		60	55	
**P3088**	3		fwd	GGGAAGCCGCTCTCTTACAC	20	95	60	60	no unintended targets under 500 bp
**P3089**			rev	GGTCTCTCCAGCACGTTGTA	20		59	55	
**P3090**	4		fwd	GGAAGCCGCTCTCTTACACG	20	76	61	60	no unintended targets under 500 bp
**P3091**			rev	TACAGGTAGTTCTGCACCCG	20		59	55	
**P3092**	5		fwd	GGGGAAGCCGCTCTCTTAC	19	89	60	63	no unintended targets under 500 bp
**P3093**			rev	CCAGCACGTTGTACAGGTAGT	21		60	52	
**P3094**	6		fwd	CACGAGTAGCCAGAGCTGC	19	103	60.5	63	no unintended targets under 500 bp
**P3095**			rev	GTGGTAGATGAACGCCCAGC	20		61	60	
**P3096**	7		fwd	TGGGGAAGCCGCTCTCTTA	19	97	60	58	no unintended targets under 500 bp
**P3097**			rev	GGTCTCTCCAGCACGTTGT	19		60	58	
**P3098**	8		fwd	CTGGGGAAGCCGCTCTCTTA	20	99	61	60	no unintended targets under 500 bp
**P3099**			rev	GGGTCTCTCCAGCACGTTG	19		60	63	
**P3100**	9		fwd	GCTCTCTTACACGAGTAGCCA	21	84	59	52	no unintended targets under 500 bp
**P3101**			rev	CTCTCCAGCACGTTGTACAGG	21		61	57	
**P3102**	1	***C9orf50***	fwd	CAAGAAGTCGGGGTCCTCC	19	80	59	63	

Primer ID	Primer pair number	Target	Primer	Primer sequence (5’-…- 3’)	Primer size / bp	Amplicon size / bp	T_m / °C	G/C content / %	Comments on *Primer-BLAST*
**P3103**			rev	AATCGGCGCCCAGGCA	16		62	69	no unintended targets under 500 bp
**P3104**	2		fwd	CAAGAAGTCGGGGTCCTCC	19	88	59	63	number of unintended targets: 3 (> 307 bp)
**P3105**			rev	GTCCCGGGAATCGGCG	16		60	75	
**P3106**	3		fwd	AAGAAGTCGGGGTCCTCCC	19	88	61	63	number of unintended targets: 2 (> 405 bp)
**P3107**			rev	CGTCCCGGGAATCGGC	16		60	75	
**P3108**	1	***SEPT9***	fwd	TGCCCAAATACAGCCTCCTG	20	70	60	55	number of unintended targets: 2 (> 365 bp)
**P3109**			rev	GCCCAGAGGGGAAGAGATTC	20		59.5	60	
**P3110**	2		fwd	GGCTGCCCAAATACAGCCT	19	119	60	58	no unintended targets under 500 bp
**P3111**			rev	AGATGTGGGCGGATGTGGAG	20		62	60	
**P3112**	3		fwd	TGTTTGGCTGCCCAAATACAG	21	70	59	48	no unintended targets under 500 bp
**P3113**			rev	GGGAAGAGATTCCTCCCCTTC	21		59	57	
**P3114**	4		fwd	TTGTTTGGCTGCCCAAATACAG	22	71	60	45	no unintended targets under 500 bp
**P3115**			rev	GGGAAGAGATTCCTCCCCTT	20		58	55	
**P3116**	5		fwd	CTGCCCAAATACAGCCTCCTG	21	116	61	57	no unintended targets under 500 bp
**P3117**			rev	GATGTGGGCGGATGTGGAGG	20		63	65	
**P3118**	6		fwd	TTGGCTGCCCAAATACAGC	19	65	59	53	no unintended targets under 500 bp
**P3119**			rev	GAAGAGATTCCTCCCCTTCCCC	22		62	59	

Table S10: Target sequences of selected DMRs used for primer design with Primer-BLAST as well as G/C content (in %) of respective target sequences. NCBI: National Center for Biotechnology Information.

Name of the DMR	Target sequence	Genomic location (NCBI accession number)	G/C content of target sequence
SEPT9	CAGAGAACTTTGTTTGGCTGCCCAAATACAGCCTCCTGCAGAAGGACCCTGCGC<br>CCGGGGAAGGGGAGGAATCTCTTCCCCTCTGGGCGCCCGCCCTCCTCGCCATGG<br>CCCGGCCTCCACATCCGCCCACATCTGGCCGCAGCGGGGCGCCCGGGGGGAGG<br>GGCTGAGGCC	NC_000017.11,<br><br>*Homo sapiens* chromosome 17, GRCh38.p14	70.20%
KCNQ5	CGCCAGGGGCGACGGCCTGCTACTGCTGGGCACCCGCGCGGCCACGCTCGGTGG<br>CGGCGGCGGTGGCCTGAGGGAGAGCCGCCGGGGCAAGCAGGGGGCCCGGATGA<br>GCCTGCTGGGGAAGCCGCTCTCTTACACGAGTAGCCAGAGCTGCCGGCGCAACG<br>TCAAGTACCGGCGGGTGCAGAACTACCTGTACAACGTGCTGGAGAGACCCCGCG<br>GCTGGGCGTTCATCTACCACGCTTTCGTGTGAGTACCCGCGCCCCCTGCTATGCC<br>C	NC_000006.11,<br><br>*Homo sapiens* chromosome 6, GRCh37.p13	70.10%
CLIP4	TGCACTGCGCGCGCGCCCACCCCGCGTGGGAGGCAGCGGGAGGGGCCCGGAGA<br>GGTGTGGAGCGGCGCGGCGGGAGGCTCCGTGGGCGGCCACGGGAGACAGCGCC<br>GGCGGGAGCGCGCCTCTCGGCCTTTCCTCCGCGCCCCCGCGTCCCCAGCCGGCC<br>GCTCCGAGAGGACCCGGAGGAGGCAGGTGGGCCGGGGGCGC	NC_000002.11,<br><br>*Homo sapiens* chromosome 2, GRCh37.p13	81.60%
C9orf50	CGCCCAAGAAGTCGGGGTCCTCCCTGGCCACGCGCCTCCGGGGGCGCTCGCGCT<br>CTCCAGGCCCTGGCTGCCTGGGCGCCGATTCCCGGGACGCGCCGGCCGACAGCA<br>GGGGAGGCGGCAGCAGGGACCGCAGCAGCCCCCGCTTCCGCACGGCCCGCCGG<br>GTCGCGGT	NC_000009.11,<br><br>*Homo sapiens* chromosome 9, GRCh37.p13	79.30%

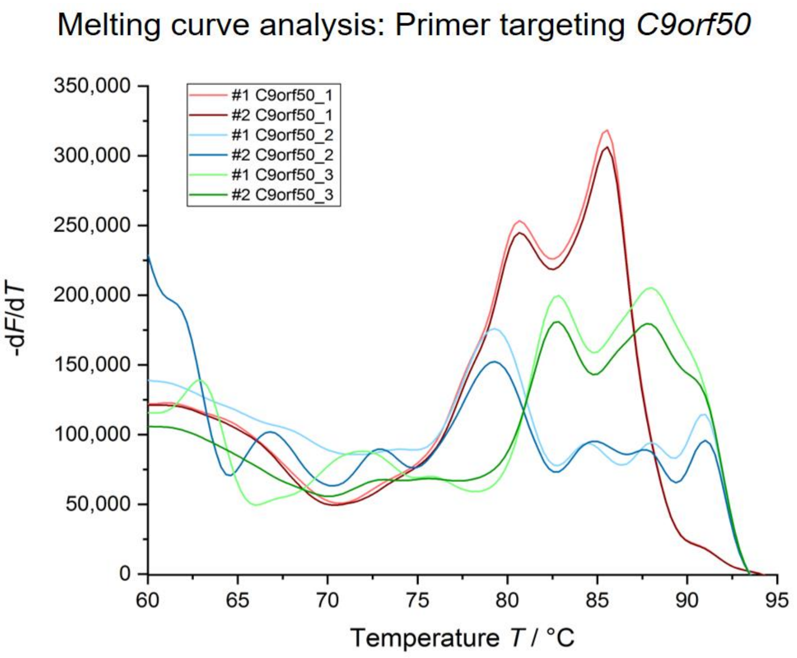

Fig. S3: Melting curve of samples containing the three primer pairs targeting C9orf50 in level 2 test (template: HCT116_ctDNA). Multiple Tm peaks are visible in melting curve for all measured samples. Technical replicates are marked with #1 and #2. Tm: melting temperature.

Table S11: (A) Evaluation of the technical sensitivity of the triplex dPCR system using a seven-point, semi-logarithmic dilution series of HCT116_ctDNA. Each dilution level was measured in triplicate to assess technical reproducibility. The table provides the mean dPCR quantification values (in cps/µl) for each target across the three channels (Blue: KCNQ5, Green: C9orf50, Red: SEPT9), along with the corresponding standard deviations (SD). To maintain a consistent DNA background, 1 ng of filler DNA was added to each sample. Concentration values that were used for linear regression are highlighted. (B) The selection of suitable data points for linear regression was based on coefficient of variation (CV) which was calculated for each data point (see formula down below). Criteria for selection were: CV under 50 % and number of positive droplets detected across all three replicates has to be greater than or equal to three.

(A)

CRC_cfDNA input	N=3	Blue_Channel_Concentration *KCNQ5* / (cps/µl)	Green_Channel_Concentration *C9orf50* / (cps/µl)	Red_Channel_Concentration *SEPT9* / (cps/µl)
0 ng (DNA background)	Mean value	0	0	0
	SD	0	0	0
0.01 ng	Mean value	0.02	0	0.02
	SD	0.04	0	0.04
0.0316 ng	Mean value	0.21	0.11	0.26
	SD	0.07	0.14	0.15
0.06 ng	Mean value	0.28	0.19	0.45
	SD	0.19	0.15	0.11
0.1 ng	Mean value	0.32	0.07	0.71
	SD	0.08	0	0.23
0.2 ng	Mean value	0.66	0.35	1.19
	SD	0.24	0.14	0.33
0.316 ng	Mean value	1.14	0.98	2.06
	SD	0.12	0.3	0.74
1 ng	Mean value	4.6	2.55	7.42
	SD	0.32	0.45	0.85

Values used for linear regression

(B)

Calculation of coefficient of variation (in %):

$$CV = \frac{\sigma}{\mu} * 100$$

σ: **standard deviation**
μ: **mean value**

mnDNA Input	Coefficient of variation (CV) for *KCNQ5* quantification (in %)	Coefficient of variation (CV) for *C9orf50* quantification (in %)	Coefficient of variation (CV) for *SEPT9* quantification (in %)
**0.01 ng**	173.21	---	173.21
**0.0316 ng**	31.48	123.63	58.2
**0.06 ng**	68.1	77.47	25.24
**0.1 ng**	23.21	0	31.54
**0.2 ng**	36.34	38.95	27.92
**0.316 ng**	10.2	31.01	36
**1 ng**	6.95	17.47	11.47

Values used for linear regression

Experimental Analysis of Buffer Effects on Droplet Formation in dPCR

The impact of cfMeDIP buffers on droplet formation in dPCR was assessed using the MagMeDIP kit's standard elution protocol. Previous measurements of Input Control (IC) samples from cfMeDIP experiments indicated a marked reduction in the number of analyzable droplets compared to Immunoprecipitated (IP) samples. Specifically, in testing IC samples, the majority showed fewer than 15,000 droplets that were analyzable. This reduction is hypothesized to result from certain residual components in the IC samples that interfere with droplet stability, leading to droplet merging and, consequently, a lower count of analyzable droplets.

Notably, the only procedural difference between IC and IP samples is the lack of a purification step for IC samples, which means that they retain remnants of MagBuffers A and B, unlike the purified IP samples. Therefore, this experiment was designed to investigate whether specific cfMeDIP buffers (MagBuffers A and B, and DIB) are responsible for the droplet-merging effect observed in IC samples.

To assess this, two buffer combinations, designated as Buffer X and Buffer Y, were prepared and tested on the Naica System for Crystal digital PCR (Stilla Technologies):

- Buffer X: This solution contained MagBuffers A and B, as well as DIB, mirroring the composition of IC samples in a standard cfMeDIP experiment. The aim was to determine if the combination of all three buffers causes droplet merging.

- Buffer Y: This solution included only MagBuffers A and B (as used in standard cfMeDIP and mirroring the composition present in IC sample) to test whether these two buffers alone could be responsible for droplet instability.

Both buffer solutions were prepared and analyzed without the addition of primers, mediator probes, or universal reporters to focus solely on droplet formation. Technical duplicates of each sample were measured to ensure reproducibility. The table below shows screenshots of Sapphire Chips illustrating the droplet generation process for samples containing Buffer X and Buffer Y. Merging of droplets was observed in samples containing Buffer X and Buffer Y, suggesting that remnants of MagBuffer A and/or B may be responsible for the observed droplet coalescence.

This experiment was crucial in the decision to add an extra purification and elution step for both IC and IP samples to eliminate the problematic MagBuffers A and B, thereby significantly increasing the amount of analyzable droplets. The IPure Kit V2 (Diagenode) is part of the published cfMeDIP-seq protocol (Shen *et al.*[1], 2019) and was therefore integrated into the cfMeDIP-dPCR protocol.

Table S12: Screenshots of Sapphire Chips of dPCR runs and number of droplets testing the different buffer compositions Buffer X and Buffer Y. Goal of the experiment was to evaluate which buffers are responsible for observed droplet merging in chips measuring IC samples.

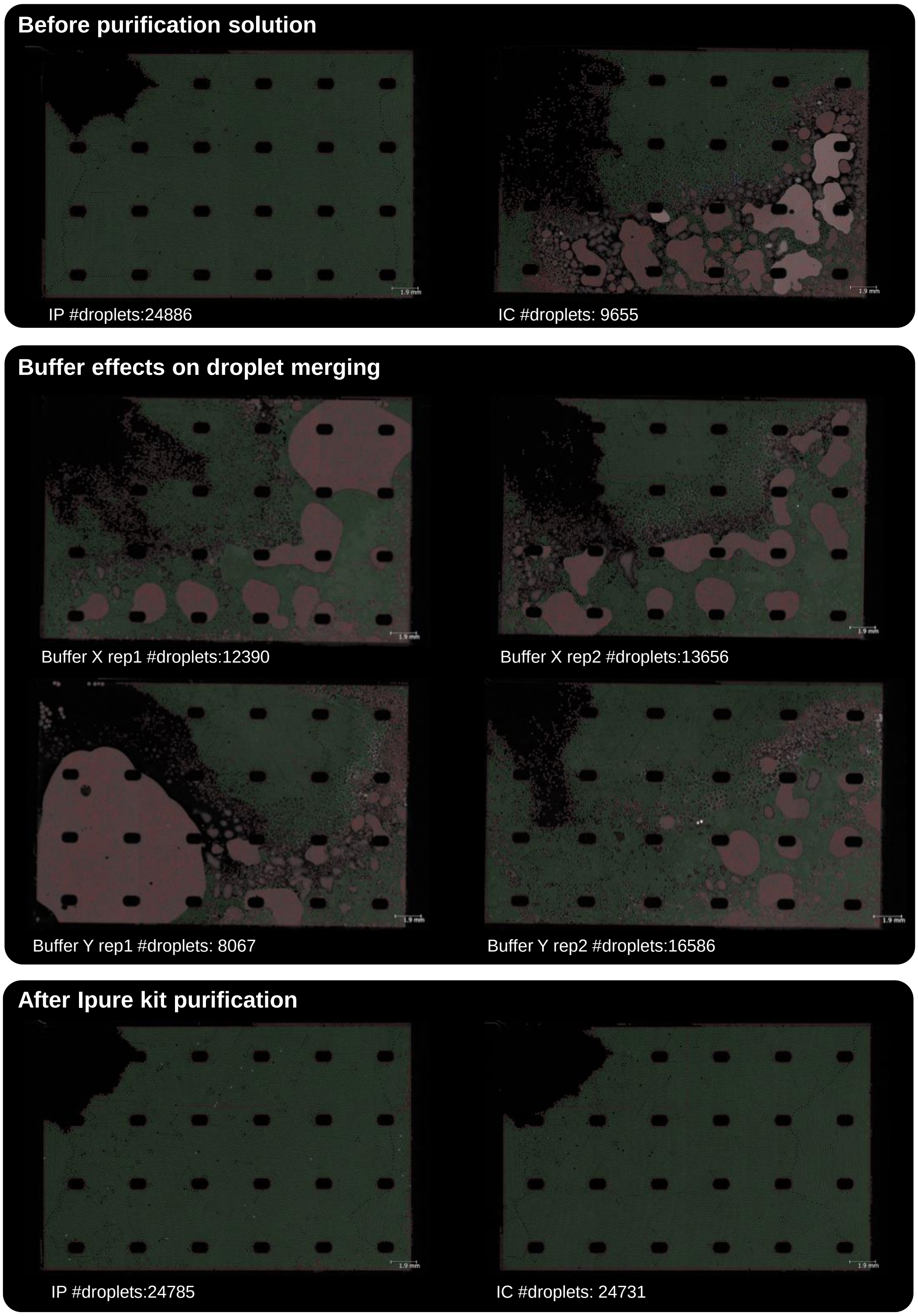

Table S13: Patient and control characteristics. EO-CRC: early-onset CRC, UICC: union internationale contre le cancer.

	EO-CRC	non-CRC
Total	32	29
Sex n (%)		
Female	15 (46.9)	18 (62.0)
Male	17 (53.1)	11 (38.0)
Age (years)		
Mean (SD)	42.8 (6.5)	46.6 (16.6)
UICC stage, n (%)		
Stage 0	2 (6.3)	-
Stage I	1 (3.1)	-
Stage II	6 (18.8)	-
Stage III	9 (28.1)	-
Stage IV	13 (40.6)	-
Unspecific or missing	1 (3.1)	
Anamnesis n (%)		
Hereditary	4 (12.5)	-
Relapse	3 (9.4)	-
Follow-up	2 (6.3)	-
Primary	22 (68.8)	-
Unspecific or missing	1 (3.1)	-

Table S14: Comparison of our cfMeDIP-dPCR approach with the sensitivity-matched bisulfite-conversion based dPCR method termed TriMeth by Jensen et al. (2019) in terms of extracted plasma per individual and calculations for effectively tested plasma volumes.

$$V_{Plasma\_tested} = V_{Plasma} * Factor_{Eluate} * Factor_{dPCR}$$

	cfMeDIP-dPCR	Bisulfite-dPCR (TriMeth)[2]	Factor
**V_Plasma**	2000 µl	16000 µl	8
V_Eluate	75 µl	100 µl	
V_Eluate_Input	53 µl	100 µl	
**Factor_Eluate**	0.71	1.00	
V_Eluate_Output	50 µl	25 µl	
V_dPCR_per_Run	9.50 µl	9 µl	
#Runs	2	2	
V_dPCR_total	19 µl	18 µl	
**Factor_dPCR**	0.38	0.72	
**V_Plasma_tested**	**537.07 µl**	**11520.00 µl**	**21**

OUTLIVE-CRC Consortium member list

Table S15: The members of the OUTLIVE-CRC Consortium, listed alphabetically, are as follows.

Larissa Almeida
Joshua Benkö
Isabell Berneburg
Hauke Busch
Nikolas von Bubnoff
Lea Christiansen
Ruth Deck
Eva Dazert-Klebsattel
Stefanie Derer-Petersen
Timo Gemoll
Clarissa Gottschild
Andreas Heidenreich
Tobias Hutzenlaub
Yumi Kaku
Alexander Katalinic
Ann-Kristin Kock-Schoppenhauer
Axel Künstner
Cassandra Lill
Regina Maushagen
Martina Oberländer
Jasmin Ostermann
Franziska Schmelter
Franziska Schulz
Christian Sina
Torsten Schröder
Judith Sum
Holger Sültmann
Sebastian Tornow
Truong-Tu Truong
Peter Juelg
Daniel Vondran

References

(1) Shen, S. Y.; Burgener, J. M.; Bratman, S. V.; Carvalho, D. D. de. Preparation of cfMeDIP-seq libraries for methylome profiling of plasma cell-free DNA. *Nat Protoc* **2019**, *14* (10), 2749–2780.

(2) Jensen, S. Ø.; Øgaard, N.; Ørntoft, M.-B. W.; Rasmussen, M. H.; Bramsen, J. B.; Kristensen, H.; Mouritzen, P.; Madsen, M. R.; Madsen, A. H.; Sunesen, K. G.; Iversen, L. H.; Laurberg, S.; Christensen, I. J.; Nielsen, H. J.; Andersen, C. L. Novel DNA methylation biomarkers show high sensitivity and specificity for blood-based detection of colorectal cancer-a clinical biomarker discovery and validation study. *Clin Epigenetics* **2019**, *11* (1), 158.
